# Simultaneous determination of multiple components in rat plasma by UPLC-MS/MS for pharmacokinetic studies after oral administration of *Pogostemon cablin* extract

**DOI:** 10.3389/fphar.2024.1293464

**Published:** 2024-05-22

**Authors:** Yameng Zhu, Huizi Ouyang, Zhenguo Lv, Guangzhe Yao, Minglei Ge, Xiunan Cao, Yanxu Chang, Jun He

**Affiliations:** ^1^ First Teaching Hospital of Tianjin University of Traditional Chinese Medicine, Tianjin, China; ^2^ National Clinical Research Center for Chinese Medicine Acupuncture and Moxibustion, Tianjin, China; ^3^ State Key Laboratory of Component-Based Chinese Medicine, Tianjin University of Traditional Chinese Medicine, Tianjin, China

**Keywords:** *Pogostemon cablin* extract, prototype components, pharmacokinetic, rat plasma, UPLC-MS/MS

## Abstract

**Introduction:** *Pogostemon cablin* (PC) is used in traditional Chinese medicine and food, as it exerts pharmacological effects, such as immune-modulatory, antibacterial, antioxidant, antitumor, and antiviral. Currently, the pharmacokinetics (PK) studies of PC mainly focus on individual components. However, research on these individual components cannot reflect the actual PK characteristics of PC after administration. Therefore, the simultaneous determination of multiple components in rat plasma using UPLC-MS/MS was used for the pharmacokinetic study after oral administration of PC extract in this study, providing reference value for the clinical application of PC.

**Methods:** In the present study, a reliable and sensitive ultra-high performance liquid chromatography/tandem mass spectrometry (UPLC-MS/MS) method was developed and validated for the simultaneous determination of 15 prototype components (vanillic acid, vitexin, verbascoside, isoacteoside, hyperoside, cosmosiin, apigenin, β-rhamnocitrin, acacetin, ombuin, pogostone, pachypodol, vicenin-2, retusin, and diosmetin-7-O-β-D-glucopyranoside) in rat plasma after oral administration of the PC extract. Plasma samples were prepared via protein precipitation using acetonitrile, and icariin was used as the internal standard (IS).

**Results:** The intra-day and inter-day accuracies ranged from −12.0 to 14.3%, and the precision of the analytes was less than 11.3%. The extraction recovery rate of the analytes ranged from 70.6−104.5%, and the matrix effects ranged from 67.4−104.8%. Stability studies proved that the analytes were stable under the tested conditions, with a relative standard deviation lower than 14.1%.

**Conclusion:** The developed method can be applied to evaluate the PK of 15 prototype components in PC extracts of rats after oral administration using UPLC-MS/MS, providing valuable information for the development and clinical safe, effective, and rational use of PC.

## 1 Introduction

Medicinal plants are natural resources with crucial medicinal value, not only for the treatment of diseases but also for the enhancement of immunity and prevention of diseases. Therefore, an in-depth study of medicinal plants is of great significance (Zhu et al., 2022; Lai et al., 2023). *Pogostemon cablin* (Blanco) Benth., is a plant of the Lamiaceae family, commonly called patchouli, or “Guanghuoxiang.” *Pogostemon cablin* (PC), is extensively distributed in the tropical and subtropical areas of Asia, and widely cultivated in the Philippines, India, and southern and southwestern China (Su et al., 2017; Chen et al., 2020). As a traditional herbal medicine, PC was used as a stomach tonic to remove dampness, improve indigestion, and relieve vomiting and diarrhea. It was also used to treat the common cold, headache, nausea, fever, and so on (Zhang et al., 2019; Chinese Pharmacopoeia Committee, 2020; Li et al., 2023). There are a number of components in PC, such as monoterpenoids, triterpenoids, sesquiterpenoids, phytosterols, and flavonoids. Studies have suggested that PC has immune-modulatory, antibacterial, antioxidant, antitumor, and antiviral bioactivities due to its multi-component properties (Kiyohara et al., 2012; Kim et al., 2015; Liu et al., 2015; Junren et al., 2021). PC is one of the ingredients in the formulations of many famous traditional Chinese patent medicines, including Huoxiang Zhengqi Oral Liquid and Huodan Wan (Pills). It can be used to treat gastrointestinal disorders with Huoxiang Zhengqi Oral Liquid, and Huodan Wan can be used for cold rhinitis and nasal congestion caused by dampness-heat in clinical (Lan and Yuan, 2018; Zhao et al., 2019). In addition to the traditional use of herbal medicine, PC also has high edible value and health benefits due to its various nutrients, including amino acids, proteins, vitamins, and minerals (Zhang, 2004). The leaves or stems of fresh PC can be stir-fried, fried, dipped in sauce, cold mixed, pickled, cooked in soup or congee, and it is mainly used as side dishes and stews to taste because of its properties of fishiness-eliminating and flavor-enhancing. Huoxiang jam is considered a food with higher health value. Meanwhile, PC is combined with *Mentha haplocalyx, Glycyrrhiza uralensis, Perilla frutescens, Ophiopogonis Radix, Chrysanthemum morifolium*, and other raw materials, which can form a variety of medicinal teas with fitness, beauty, and disease treatment effects. Moreover, PC can be used as a fixing agent for various perfumes, and as a raw material for cosmetics and oral hygiene products (Huo et al., 2007; Zhang J. et al., 2016; Sandes et al., 2016).

Pharmacokinetics (PK) is the study of the absorption, distribution, metabolism, and excretion of drugs *in vivo*, which plays an important role in the determination of the drug’s kinetics and bioavailability. Notably, PK is very important for evaluating the absorption properties of different active components of traditional Chinese medicine (TCM). Moreover, the development of sensitive and reliable biological sample analysis technology to simultaneously determine multiple active ingredients *in vivo* is a hotspot in the PK research of TCM extracts, due to the complex composition and significant differences in the content (Cao et al., 2021). Therefore, an accurate and selective bioanalytical method is urgently required for the simultaneous determination of multiple biological components in plasma to understand the characteristics and diversity of the PK properties of PC.

In this study, a method for the simultaneous detection of 15 compounds (vanillic acid, vitexin, verbascoside, isoacteoside, hyperoside, cosmosiin, apigenin, β-rhamnocitrin, acacetin, ombuin, pogostone, pachypodol, diosmetin-7-O-β-D-glucopyranoside, vicenin-2, and retusin) in rat plasma was established and validated using ultra-high-performance liquid chromatography/tandem mass spectrometry (UPLC-MS/MS), which provided a reference for the clinical safe, effective and rational use of PC.

## 2 Materials and methods

### 2.1 Chemicals and reagents

Standard compound including vanillic acid, vitexin, verbascoside, isoacteoside, hyperoside, cosmosiin, apigenin, β-rhamnocitrin, acacetin, ombuin, pogostone, pachypodol, diosmetin-7-O-β-D-glucopyranoside, vicenin-2, and retusin (purity ≥98%) were purchased from Chengdu Desite Bio-Technology Co., Ltd. (Chengdu, China). Icariin [internal standard (IS)] was provided by Tianjin Yifang Zhongkang Pharmaceutical Technology Co., Ltd. (Tianjin, China). Fisher Scientific (Fair Lawn, NJ, United States) supplied the chromatographic grade methanol (ME) and acetonitrile (ACN). The chromatographic grade of formic acid (FA) was provided by ROE (St Louis, MO, United States). PC was purchased from Guangdong province (China).

### 2.2 Chromatography and mass spectrometry (MS)

The analyte was separated and detected through the utilization of a UPLC-MS/MS system, consisting primarily of the Agilent-1290 high-performance liquid chromatography system (Agilent, United States) and the Agilent-6470 triple quadrupole tandem mass spectrometer (Agilent, United States).

The components were separated using an ACQUITY UPLC®BEH C18 column (2.1 × 100 mm, 1.7 µm) maintained at a temperature of 25°C. 0.1% FA-water and ACN were used as mobile phases A and B. The gradient elution conditions were as follows: 0–4 min, 14%–53% B; 4–5 min, 53%–100% B; and 5–7 min, 100%–100% B. A flow rate of 0.3 mL/min and an injection volume of 5 μL were used.

Mass spectrometry analysis was conducted in the multiple reaction monitoring (MRM) mode with both positive and negative ionization (Zhu et al., 2022). The parameters for the mass spectrometer included drying gas (N_2_) temperature, gas flow rate, atomizer pressure, and capillary voltage of 320°C, 7 L/min, 35 psi, and 3500 V, respectively. The detailed MRM parameters can be found in Supplementary Table S1.

### 2.3 PC extract preparation

PC (0.5 kg) was accurately weighed and extracted with 10-fold ethanol (85%, *v/v*) under heated reflux conditions twice for 1.5 h. Next, the solutions for extraction underwent filtration and were subsequently combined. The ensuing mixture underwent concentration through evaporation under decreased pressure. Ultimately, the dried PC extract was stored in a dry environment for further analysis. The levels of vanillic acid, vitexin, verbascoside, isoacteoside, hyperoside, cosmosiin, apigenin, β-rhamnocitrin, acacetin, ombuin, pogostone, pachypodol, vicenin-2, retusin, and diosmetin-7-O-β-D-glucopyranoside in the PC extract are displayed in Supplementary Table S2.

### 2.4 Preparation of standard solutions, calibration standards, and quality control (QC) samples

Vanillic acid, vitexin, verbascoside, isoacteoside, hyperoside, cosmosiin, apigenin, β-rhamnocitrin, acacetin, ombuin, pogostone, pachypodol, vicenin-2, retusin, diosmetin-7-O-β-D-glucopyranoside, and icariin (IS) were individually weighed and mixed with ME to prepare standard stock solution (1 mg/mL). To obtain the subsequent working solutions, the standard stock solutions were diluted using ME.

To prepare the calibration solutions, 20 μL of a mixture of working solution and IS was added to blank rat plasma (100 μL), resulting in concentrations: 20, 50, 100, 200, 400, 1,000, 2000, 4,000, and 8,000 ng/mL for vanillic acid and vicenin-2; 0.5, 1.25, 2.5, 5, 10, 25, 50, 100, and 200 ng/mL for vitexin, cosmosiin, and ombuin; 0.3, 0.75, 1.5, 3, 6, 15, 30, 60, and 120 ng/mL for hyperoside, β-rhamnocitrin, acacetin, and diosmetin-7-O-β-D-glucopyranoside; 10, 25, 50, 100, 200, 500, 1,000, 2000, and 4,000 ng/mL for verbascoside; 2, 5, 10, 20, 40, 100, 200, 400, and 800 ng/mL for isoacteoside; 1.5, 3.75, 7.5, 15, 30, 75, 150, 300, and 600 ng/mL for apigenin; 1, 2.5, 5, 10, 20, 50, 100, 200, and 400 ng/mL for pachypodol; 0.2, 0.5, 1, 2, 4, 10, 20, 40, and 80 ng/mL for retusin; 125, 312.5, 635, 1,250, 2,500, 6,250, 12,500, 25,000, and 50,000 ng/mL for pogostone. QC samples at three concentrations (low, medium, and high) were obtained similarly. Prior to analysis, all solutions were stored in an environment of 4°C.

### 2.5 Preparation of plasma sample

The 100 μL plasma sample was combined with 20 μL of ME, 20 μL of IS (500 ng/mL), and 2.5 μL of FA, followed by vortexing for 1 min. This mixture underwent extraction with 600 μL ACN through vortexing at room temperature for 3 min. Subsequent to centrifugation at 14,000 rpm for a period of 10 min, the resulting supernatant was moved to another tube and dried under a stream of nitrogen. The dried residue was dissolved in 100 μL ME-ACN (50:50 *v/v*), followed by vortexing for 5 min, and subsequent centrifugation at 14,000 rpm for 10 min. Finally, 20 μL of the supernatant was used for analysis.

### 2.6 Method validation

#### 2.6.1 Specificity

In order to assess specificity, chromatograms of rat plasma samples without any added analytes, plasma with added analytes, and post-dosing plasma samples from rats following oral ingestion of PC were analyzed.

#### 2.6.2 Linearity and LLOQ

By graphing the correlation between the peak area ratios of each component to IS versus the concentration of those particular analytes to obtain calibration curves. The regression association was depicted utilizing a linear formula with a weighting factor of 1/*x* ^2^. The determination of LLOQ relied on the baseline noise, ensuring a signal-to-noise ratio of around ten.

#### 2.6.3 Precision and accuracy

QC samples three concentrations (20, 400, and 6,400 ng/mL for vanillic acid and vicenin-2; 0.5, 10, and 160 ng/mL for vitexin, cosmosiin, and ombuin; 0.3, 6, and 96 ng/mL for hyperoside, β-rhamnocitrin, acacetin and diosmetin-7-O-β-D-glucopyranoside; 10, 200, and 3200 ng/mL for verbascoside; 2, 40, and 640 ng/mL for isoacteoside; 1.5, 30, and 480 ng/mL for apigenin; 1, 20, and 320 ng/mL for pachypodol; 0.2, 4, and 64 ng/mL for retusin; 125, 2,500, and 40,000 ng/mL for pogostone) were analyzed six repetitions over one or three consecutive days to assess the precision and accuracy. Relative error (RE) was used to determine accuracy, while the precisions were expressed as the relative standard deviation (RSD).

#### 2.6.4 Extraction recovery and matrix effect

Analyzing the peak areas of QC samples and the peak areas of the post-extraction mixed samples to measure the extraction recovery rates at three concentrations. Matrix effects were determined by comparing the peak areas of QC samples in post-extracted mixed samples to those in standard solutions, calculating the ratio for evaluation.

#### 2.6.5 Stability

Plasma samples were retained for 12 h in an auto-sampler, exposed to room temperature for 4 h, undergoing three freeze-thaw cycles, and stored at −80°C for a period of 1 week to assess the stability of all compounds.

### 2.7 PK study

Six male rats (220 ± 10 g) were utilized in this experiment and were not restricted from drinking water but fasted for 12 h before the investigation. A concentration of 0.5 g/mL of PC extract was obtained by dissolving in 0.5% CMC-Na aqueous solution. The PC suspension of the 4.0 g/kg dose was given to rats by single oral administration. Samples of blood (300 μL) were obtained from the orbital venous plexus prior to and following oral dosing at 0, 0.03, 0.08, 0.25, 0.5, 0.75, 1, 2, 6, 10, 12, 24, 36, and 48 h. Subsequently, the plasma collected underwent centrifugation at a speed of 7000 rpm for a duration of 10 min. It was then moved to clean tubes and kept at a temperature of −80°C.

### 2.8 Data analysis

Data in this study was presented as the mean ± standard deviation (SD). The MassHunter Workstation software (version B.09.00, Agilent, United States) was utilized for determining the plasma levels of the 15 analytes. Additionally, PK parameters were analyzed using DAS 3.0 Software (Medical College of Wannan, China).

## 3 Result and discussion

### 3.1 Optimization of chromatography and MS

To improve the separation of the 15 compounds, an investigation was conducted on the impact of different columns, including ACQUITY UPLC BEH C18 (2.1 × 100 mm, 1.7 µm) and ACQUITY UPLC HSS T3 (2.1 × 100 mm, 1.8 µm), on the chromatographic peaks and retention times. The findings revealed that the ACQUITY UPLC BEH C18 (2.1 × 100 mm, 1.7 µm) column provided superior separation capability for the 15 compounds. Different mobile phases were also evaluated, such as 0.1% FA/water-ACN, 0.1% FA/water-ME, water-ME, and water-ACN, to optimize the separation of all compounds. The findings demonstrated that the 0.1% FA/water-ACN proved to be superior in improving the separation and peak profiles of the compounds. All 15 components and IS were eluted successfully within a 7-min, and with no observed interference peaks.

Optimizing the primary parameters of the MS was crucial in enhancing the response of compounds. In positive ion mode, the compounds retusin, diosmetin-7-O-β-D-glucopyranoside, and IS were examined, while others were assessed in negative ion mode.

### 3.2 Sample preparation

The study evaluated four different extraction methods to determine the most effective method for preparing plasma samples, including protein precipitation using ME or ACN, ethyl acetate liquid-liquid extraction, and extraction using a mixture of ME and ACN (*v/v* = 1:4). The findings indicated that the protein precipitation using ACN showed higher extraction recovery rates for the 15 analytes tested. Additionally, different volumes (400, 600, 800, and 1,000 μL) of precipitated solvent were used to assess the extraction recovery and matrix effect. The outcomes demonstrated that the extraction recovery and matrix effects of the ACN-protein precipitation using 600 μL satisfied the criteria for analyzing biological specimens, with no interference from endogenous compounds. Additionally, the impacts of various reconstitution solvents such as ME, ACN, 50% ME, and ME-ACN (*v/v* = 1:1, 1:4, and 4:1) were assessed. The results indicated that the redissolution effectiveness was optimal with a ME-ACN mixture of (*v/v* = 1:1).

### 3.3 Method validation

#### 3.3.1 Specificity

**FIGURE 1:**
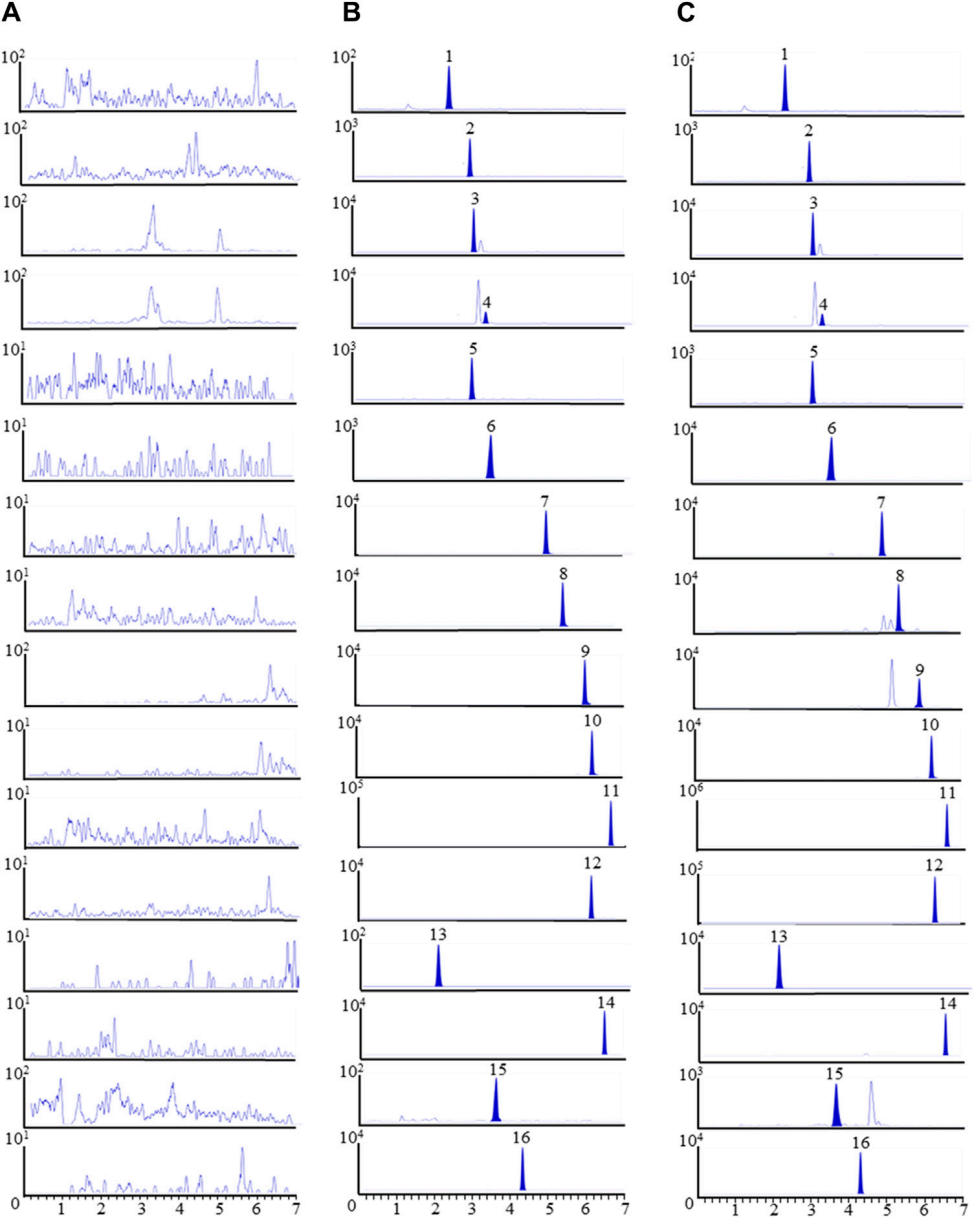
The MRM chromatograms of 15 components and IS in rat plasma samples. **(A)** Plasma samples without any added analytes, **(B)** plasma samples combined with the 15 compounds and IS, **(C)** post-dosing plasma samples from rats following oral ingestion of PC. 1. Vanillic acid, 2. Vitexin, 3. Verbascoside, 4. Isoacteoside, 5. Hyperoside, 6. Cosmosiin, 7. Apigenin, 8. β-Rhamnocitrin, 9. Acacetin, 10. Ombuin, 11. Pogostone, 12. Pachypodol, 13. Vicenin-2, 14. Retusin 15. Diosmetin-7-O-β-D-glucopyranoside 16. Icariin (IS).

The MRM chromatograms in Figure 1 displayed the rat plasma samples without any added analytes (A), plasma samples combined with the 15 compounds and IS (B), and post-dosing plasma samples from rats following oral ingestion of PC (C). The results suggested no noticeable interference peaks were noted at the retention time of the 15 compounds and IS.

#### 3.3.2 Linearity and LLOQ

The calibration curves, linear ranges, correlation coefficients, and LLOQ for 15 compounds are shown in Table 1. Within their respective concentration ranges, the calibration profiles of these 15 compounds exhibited excellent linearity (*r* > 0.996). The LLOQ of vanillic acid, vitexin, verbascoside, isoacteoside, hyperoside, cosmosiin, apigenin, β-rhamnocitrin, acacetin, ombuin, pogostone, pachypodol, diosmetin-7-O-β-D-glucopyranoside, vicenin-2, and retusin and were 20.0, 0.5, 10.0, 2.0, 0.3, 0.5, 1.5, 0.3, 0.3, 0.5, 30.0, 1.0, 0.3, 20.0, and 0.2 ng/mL, respectively.

**TABLE 1:** Standard curve regression equation, linear range and LLOQ of 15 components.

Compound	Calibration curve	Linear range (ng/mL)	Correlation coefficients (r)	LLOQ (ng/mL)
Vanillic acid	*Y* = 0.0023X − 2.3543^E-004^	20–8,000	0.998	20.0
Vitexin	*Y* = 0.0919X + 1.3292^E-004^	0.5–200	0.998	0.5
Verbascoside	*Y* = 0.0335X + 0.0024	10–4,000	0.999	10.0
Isoacteoside	*Y* = 0.0490X + 0.0015	2–800	0.997	2.0
Hyperoside	*Y* = 0.0687X − 1.3570^E-004^	0.3–120	0.998	0.3
Cosmosiin	*Y* = 0.0.1527X + 0.0031	0.5–200	0.998	0.5
Apigenin	*Y* = 0.0805X + 0.0013	1.5–600	0.999	1.5
β-rhamnocitrin	*Y* = 0.2143X + 0.0057	0.3–120	0.999	0.3
Acacetin	*Y* = 0.4803X + 0.0023	0.3–120	0.998	0.3
Ombuin	*Y* = 0.0472X + 0.0015	0.5–200	0.996	0.5
Pogostone	*Y* = 0.0056X + 0.1677	125–50000	0.999	30.0
Pachypodol	*Y* = 0.2386X + 0.0209	1–400	0.999	1.0
Vicenin-2	*Y* = 0.0112X − 0.0013	20–8,000	0.997	20.0
Retusin	*Y* = 0.0989X + 0.0070	0.2–80	0.996	0.2
Diosmetin-7-O-β-D-glucopyranoside	*Y* = 0.2742X − 4.2961^E-004^	0.3–120	0.999	0.3

#### 3.3.3 Precision and accuracy

The precision and accuracy of QC samples at three concentrations are provided in Table 2. The RSD of intra- and inter-day were below 11.3%, the RE of intra-day ranged from −12.0%–14.3%, and the inter-day ranged from −6.5%–13.2%. These findings indicated that is accurate and precise to a satisfactory degree.

**TABLE 2:** Accuracy and precision of 15 components in rat plasma (*n* = 6).

Compound	Spiked concent-ration (ng/mL)	Intra-day			Inter-day		
		Measured (ng/mL)	RE (%)	RSD (%)	Measured (ng/mL)	RE (%)	RSD (%)
Vanillic acid	20	22.3 ± 0.5	11.5	2.4	22.1 ± 0.6	10.6	2.5
	400	401.0 ± 6.1	0.3	1.5	395.5 ± 15.4	−1.1	3.9
	6,400	6,544.7 ± 92.7	2.3	1.4	6,203.9 ± 375.4	−3.1	6.1
Vitexin	0.5	0.6 ± 0.1	12.0	10.5	0.6 ± 0.1	11.5	8.5
	10	9.8 ± 0.5	−1.7	5.1	10.3 ± 0.3	2.7	3.1
	160	153.7 ± 3.9	−3.9	2.5	168.3 ± 11.7	5.2	7.0
Verbascoside	10	11.4 ± 1.2	14.3	10.6	11.1 ± 0.3	10.8	2.8
	200	193.8 ± 15.8	−3.1	8.2	193.4 ± 13.1	−3.3	6.7
	3200	3147.6 ± 156.6	−1.6	5.0	3498.1 ± 208.3	9.3	6.0
Isoacteoside	2	2.3 ± 0.2	12.4	7.6	2.2 ± 0.2	9.8	7.4
	40	43.9 ± 2.1	9.8	4.7	43.7 ± 2.1	9.3	4.8
	640	657.2 ± 27.8	2.7	4.2	661.9 ± 21.4	3.4	3.2
Hyperoside	0.3	0.3 ± 0.1	12.1	10.9	0.3 ± 0.0	3.9	2.3
	6	6.1 ± 0.3	1.2	5.5	6.1 ± 0.2	1.9	3.6
	96	96.4 ± 2.0	0.4	2.1	104.6 ± 6.5	9.0	6.2
Cosmosiin	0.5	0.5 ± 0.0	3.2	2.0	0.5 ± 0.0	3.3	2.5
	10	10.0 ± 0.4	0.3	3.6	10.2 ± 0.3	2.4	2.5
	160	168.8 ± 5.5	5.5	3.3	164.8 ± 4.4	3.0	2.6
Apigenin	1.5	1.7 ± 0.2	13.4	9.3	1.7 ± 0.2	12.1	9.9
	30	29.4 ± 1.6	−1.9	5.4	31.8 ± 1.5	6.0	4.8
	480	484.8 ± 8.7	1.0	1.8	487.8 ± 5.0	1.6	1.0
β-rhamnocitrin	0.3	0.3 ± 0.1	14.0	9.2	0.3 ± 0.1	12.6	9.3
	6	5.3 ± 0.5	−12.0	8.5	6.1 ± 0.2	0.8	3.4
	96	87.2 ± 9.3	−9.2	10.6	100.8 ± 4.0	5.0	4.0
Acacetin	0.3	0.3 ± 0.0	10.8	11.3	0.3 ± 0.1	9.2	10.2
	6	5.6 ± 0.2	−6.6	3.8	5.9 ± 0.5	−1.0	8.0
	96	95.2 ± 2.7	−0.9	2.8	97.6 ± 2.7	1.7	2.8
Ombuin	0.5	0.5 ± 0.0	6.3	6.0	0.5 ± 0.1	7.5	11.2
	10	9.8 ± 0.5	−2.3	5.0	9.5 ± 0.6	−5.1	5.9
	160	151.4 ± 4.7	−5.4	3.1	153.5 ± 1.9	−4.0	1.2
Pogostone	125	132.5 ± 7.3	6.0	5.5	131.7 ± 3.6	5.4	2.7
	2,500	2,611.9 ± 125.9	4.5	4.8	2,535.6 ± 75.2	1.4	3.0
	40,000	37,526.9 ± 1,130.5	−6.2	3.0	37,411.8 ± 1754.1	−6.5	4.7
Pachypodol	1	1.1 ± 0.1	9.5	4.9	1.1 ± 0.1	7.2	7.8
	20	22.2 ± 0.8	10.7	3.6	21.0 ± 1.1	4.8	5.4
	320	346.8 ± 13.8	8.4	4.0	317.9 ± 24.5	−0.7	7.7
Vicenin-2	20	22.7 ± 0.8	13.5	3.5	22.7 ± 1.2	13.2	5.3
	400	369.3 ± 16.7	−7.7	4.5	407.5 ± 21.8	1.9	5.3
	6,400	6,403.5 ± 115.5	0.1	1.8	6,480.6 ± 158.8	1.3	2.5
Retusin	0.2	0.2 ± 0.0	10.6	6.1	0.2 ± 0.0	6.6	4.2
	4	4.4 ± 0.1	9.5	2.9	4.2 ± 0.1	5.9	2.7
	64	64.6 ± 4.1	0.9	6.3	64.2 ± 4.2	0.3	6.5
Diosmetin-7-O-β-D-glucopyranoside	0.3	0.3 ± 0.0	13.6	5.6	0.3 ± 0.0	12.2	5.8
	6	6.19 ± 0.2	3.1	3.8	6.0 ± 0.4	−0.9	5.9
	96	102.7 ± 4.9	7.0	4.7	99.3 ± 4.3	3.4	4.3

#### 3.3.4 Extraction recovery and matrix effect

As listed in Table 3, the 15 analytes and the IS displayed extraction recovery and matrix effects within the ranges of 70.6%–104.5%, and 67.4%–104.8%, respectively. These findings revealed that the method’s extraction recovery and matrix effects were accurate and satisfactory.

**TABLE 3:** Extraction recovery and matrix effects of 15 components in rat plasma (*n* = 6).

Compound	Spiked concentration (ng/mL)	Extraction recovery (%)	RSD (%)	Matrix effect (%)	RSD (%)
Vanillic acid	20	90.2 ± 8.0	8.8	89.3 ± 7.0	7.9
	400	97.1 ± 6.8	7.0	93.1 ± 8.1	8.7
	6,400	92.1 ± 4.5	4.9	86.5 ± 8.3	9.6
Vitexin	0.5	88.1 ± 9.1	10.3	83.7 ± 6.7	7.9
	10	89.2 ± 10.3	11.5	85.1 ± 8.1	9.5
	160	76.20 ± 5.9	7.7	75.2 ± 7.4	9.8
Verbascoside	10	89.0 ± 5.4	6.0	81.2 ± 6.8	8.4
	200	92.3 ± 9.1	9.9	82.4 ± 5.78	7.0
	3200	84.9 ± 4.9	5.8	85.2 ± 7.3	8.6
Isoacteoside	2	83.1 ± 6.5	7.8	80.3 ± 8.4	10.4
	40	86.7 ± 6.8	7.9	81.8 ± 9.2	11.2
	640	91.7 ± 10.4	11.4	86.9 ± 9.3	10.7
Hyperoside	0.3	79.4 ± 6.6	8.3	88.2 ± 9.4	10.6
	6	77.3 ± 4.3	5.6	85.7 ± 7.3	8.5
	96	78.4 ± 4.4	5.6	70.7 ± 3.5	5.0
Cosmosiin	0.5	82.4 ± 7.7	9.3	91.9 ± 8.5	9.3
	10	81.0 ± 7.5	9.3	97.0 ± 6.2	6.3
	160	87.2 ± 11.2	12.8	83.1 ± 3.5	4.3
Apigenin	1.5	95.3 ± 6.9	7.3	103.3 ± 2.8	2.7
	30	81.8 ± 1.6	1.9	100.5 ± 3.0	3.0
	480	90.4 ± 4.9	5.4	83.8 ± 3.4	4.1
β-rhamnocitrin	0.3	96.0 ± 6.5	6.8	80.4 ± 4.2	5.3
	6	84.0 ± 5.5	6.6	97.8 ± 7.6	7.7
	96	86.2 ± 4.2	4.9	93.9 ± 4.3	4.6
Acacetin	0.3	94.3 ± 7.6	8.0	72.2 ± 4.8	6.7
	6	90.1 ± 7.7	8.5	92.5 ± 10.2	11.0
	96	89.0 ± 11.3	12.7	71.2 ± 9.1	12.8
Ombuin	0.5	70.6 ± 7.0	9.9	79.4 ± 9.3	11.7
	10	79.4 ± 3.0	3.8	79.1 ± 10.9	13.8
	160	88.4 ± 8.0	9.0	83.4 ± 4.7	5.6
Pogostone	125	87.4 ± 11.3	13.0	98.5 ± 12.2	12.4
	2,500	87.8 ± 5.4	6.2	99.1 ± 4.1	4.1
	40,000	78.5 ± 7.0	8.9	87.1 ± 9.7	11.1
Pachypodol	1	89.4 ± 10.5	11.7	69.3 ± 6.1	8.7
	20	104.5 ± 9.6	9.2	78.1 ± 7.0	9.0
	320	96.3 ± 9.1	9.5	82.1 ± 10.6	12.9
Vicenin-2	20	93.4 ± 5.1	5.4	89.5 ± 10.4	11.7
	400	71.1 ± 3.4	4.8	104.8 ± 13.2	12.6
	6,400	79.2 ± 10.2	12.9	76.9 ± 6.6	8.6
Retusin	0.2	95.2 ± 8.1	8.5	70.9 ± 6.3	8.9
	4	89.1 ± 11.5	12.9	69.9 ± 7.4	10.5
	64	71.6 ± 9.3	13.0	67.4 ± 6.9	10.3
Diosmetin-7-O-β-D-glucopyranoside	0.3	85.5 ± 7.0	8.1	75.8 ± 9.7	12.7
	6	89.7 ± 7.1	7.9	68.7 ± 3.9	5.7
	96	93.5 ± 5.7	6.1	72.8 ± 6.6	9.0
Icariin (IS)	500	86.2 ± 2.4	2.8	87.5 ± 2.1	2.5

#### 3.3.5 Stability

As shown in Table 4, the RSD values for the stability of all analytes tested were less than 14.1%, indicating that they were adequately stable across different conditions. This further suggested that the established UPLC-MS/MS method could effectively measure the 15 components in rat plasma.

**TABLE 4:** Stability of 15 components in rat plasma (*n* = 6).

Compound	Spiked concentration (ng/mL)	Room temperature for 4 h		Autosampler for 12 h		Three freeze-thaw cycles		−80 °C for 7 days	
		Measured (ng/mL)	RSD (%)	Measured (ng/mL)	RSD (%)	Measured (ng/mL)	RSD (%)	Measured (ng/mL)	RSD (%)
Vanillic acid	20	22.0 ± 0.4	2.0	22.7 ± 1.0	4.3	22.7 ± 0.5	2.1	23.1 ± 0.8	3.4
	400	405.6 ± 17.8	4.4	396.6 ± 8.7	2.2	374.6 ± 10.2	2.7	392.0 ± 8.5	2.2
	6,400	5821.2 ± 241.2	4.1	6,602.7 ± 196.9	3.0	6,149.2 ± 382.8	6.2	5892.7 ± 220.9	3.7
Vitexin	0.5	0.5 ± 0.0	2.0	0.5 ± 0.1	7.0	0.5 ± 0.0	3.0	0.6 ± 0.1	7.1
	10	9.7 ± 0.2	2.2	9.9 ± 0.4	4.2	9.3 ± 0.4	4.8	9.0 ± 0.4	4.0
	160	153.8 ± 6.7	4.4	166.2 ± 6.9	4.1	154.6 ± 8.7	5.6	147.4 ± 10.7	7.2
Verbascoside	10	11.3 ± 0.7	5.9	11.7 ± 1.0	8.1	11.3 ± 0.7	6.3	11.6 ± 0.8	6.9
	200	188.8 ± 4.7	2.5	197.3 ± 14.2	7.2	178.9 ± 9.6	5.4	180.0 ± 4.9	2.7
	3200	3280.8 ± 140.4	4.3	3327.3 ± 190.1	5.7	3240.3 ± 93.8	2.9	3114.3 ± 67.0	2.2
Isoacteoside	2	2.2 ± 0.1	5.7	2.2 ± 0.1	5.0	2.2 ± 0.1	3.9	2.2 ± 0.1	4.0
	40	41.6 ± 1.3	3.2	43.8 ± 1.4	3.3	43.0 ± 1.2	2.8	42.2 ± 0.9	2.2
	640	657.1 ± 21.8	3.3	666.7 ± 12.2	1.8	667.9 ± 11.3	1.7	654.5 ± 18.0	2.8
Hyperoside	0.3	0.3 ± 0.1	10.9	0.3 ± 0.0	6.5	0.3 ± 0.0	5.9	0.3 ± 0.0	2.7
	6	5.5 ± 0.4	7.6	6.0 ± 0.2	3.8	5.9 ± 0.4	6.1	5.9 ± 0.2	3.5
	96	94.9 ± 5.8	6.2	99.1 ± 4.5	4.6	95.8 ± 3.3	3.5	93.3 ± 5.3	5.6
Cosmosiin	0.5	0.5 ± 0.0	6.9	0.5 ± 0.0	3.7	0.6 ± 0.1	7.4	0.5 ± 0.0	4.2
	10	9.2 ± 0.5	5.5	10.2 ± 0.3	2.7	10.1 ± 0.5	4.5	9.6 ± 0.5	5.3
	160	163.7 ± 5.5	3.4	175.3 ± 5.9	3.4	168.0 ± 6.0	3.6	156.0 ± 2.3	1.5
Apigenin	1.5	1.6 ± 0.1	4.6	1.7 ± 0.2	9.1	1.6 ± 0.0	1.1	1.8 ± 0.1	3.7
	30	28.3 ± 0.9	3.1	31.6 ± 1.0	3.1	28.8 ± 1.2	4.3	29.0 ± 0.7	2.5
	480	478.3 ± 18.4	3.8	494.4 ± 11.3	2.3	461.1 ± 23.0	5.0	440.2 ± 27.4	6.2
β-rhamnocitrin	0.3	0.3 ± 0.0	2.5	0.3 ± 0.0	5.0	0.3 ± 0.0	4.6	0.3 ± 0.0	6.8
	6	6.0 ± 0.1	2.0	6.2 ± 0.1	1.7	5.5 ± 0.3	6.0	5.9 ± 0.2	3.4
	96	102.6 ± 4.6	4.5	103.9 ± 4.2	4.0	97.0 ± 3.3	3.4	96.2 ± 4.4	4.6
Acacetin	0.3	0.4 ± 0.1	10.5	0.3 ± 0.0	8.9	0.3 ± 0.0	5.1	0.3 ± 0.0	2.4
	6	5.4 ± 0.4	7.3	5.8 ± 0.5	8.5	5.6 ± 0.3	4.9	5.8 ± 0.2	2.7
	96	94.4 ± 1.3	1.4	96.5 ± 4.2	4.3	94.3 ± 1.4	1.5	93.5 ± 1.8	1.9
Ombuin	0.5	0.5 ± 0.0	2.6	0.5 ± 0.0	2.6	0.6 ± 0.0	7.7	0.5 ± 0.0	5.6
	10	9.9 ± 0.3	2.7	9.6 ± 0.2	2.5	9.0 ± 0.2	2.6	9.3 ± 0.4	4.5
	160	151.0 ± 5.3	3.5	155.3 ± 4.2	2.7	154.7 ± 7.3	4.7	154.9 ± 4.8	3.1
Pogostone	125	132.6 ± 8.9	6.7	131.4 ± 5.9	4.5	132.1 ± 7.6	5.7	132.8 ± 10.2	7.7
	2,500	2,756.7 ± 171.1	6.2	2,482.5 ± 168.7	6.8	2,618.0 ± 112.1	4.3	2,618.0 ± 112.1	4.3
	40,000	38,298.2 ± 647.9	1.7	37047.4 ± 2,580.0	7.0	36825.8 ± 1555.6	4.2	38116.7 ± 1,540.2	4.0
Pachypodol	1	1.1 ± 0.1	10.0	1.1 ± 0.1	4.5	1.18 ± 0.12	10.4	1.1 ± 0.1	8.3
	20	21.4 ± 2.0	9.1	18.7 ± 0.8	4.5	20.6 ± 1.7	8.3	20.8 ± 1.8	8.9
	320	337.2 ± 12.6	3.7	334.8 ± 21.1	6.3	345.1 ± 7.2	2.1	330.2 ± 21.4	6.5
Vicenin-2	20	22.4 ± 0.4	1.9	23.2 ± 0.6	2.7	23.0 ± 1.1	4.9	22.7 ± 0.3	1.2
	400	399.2 ± 2.7	0.7	409.4 ± 12.1	3.0	389.6 ± 17.1	4.4	400.1 ± 5.7	1.4
	6,400	6,476.6 ± 162.3	2.5	6,139.7 ± 284.7	4.6	6,473.2 ± 140.7	2.2	6,317.8 ± 84.9	1.3
Retusin	0.2	0.2 ± 0.0	6.8	0.2 ± 0.0	12.9	0.2 ± 0.0	7.1	0.3 ± 0.1	14.1
	4	4.1 ± 0.2	4.6	3.7 ± 0.3	9.2	4.1 ± 0.1	3.0	3.9 ± 0.4	10.2
	64	61.2 ± 1.6	2.6	69.4 ± 6.4	9.2	63.5 ± 1.8	2.8	63.3 ± 5.3	8.4
Diosmetin-7-O-β-D-glucopyranoside	0.3	0.3 ± 0.0	3.3	0.3 ± 0.0	6.4	0.3 ± 0.0	5.4	0.4 ± 0.1	10.2
	6	6.5 ± 0.2	2.9	6.3 ± 0.2	3.8	6.6 ± 0.4	5.7	6.6 ± 0.6	8.8
	96	98.0 ± 1.7	1.7	99.4 ± 4.7	4.8	103.3 ± 2.9	2.8	102.2 ± 3.6	3.5

### 3.4 PK study

**FIGURE 2:**
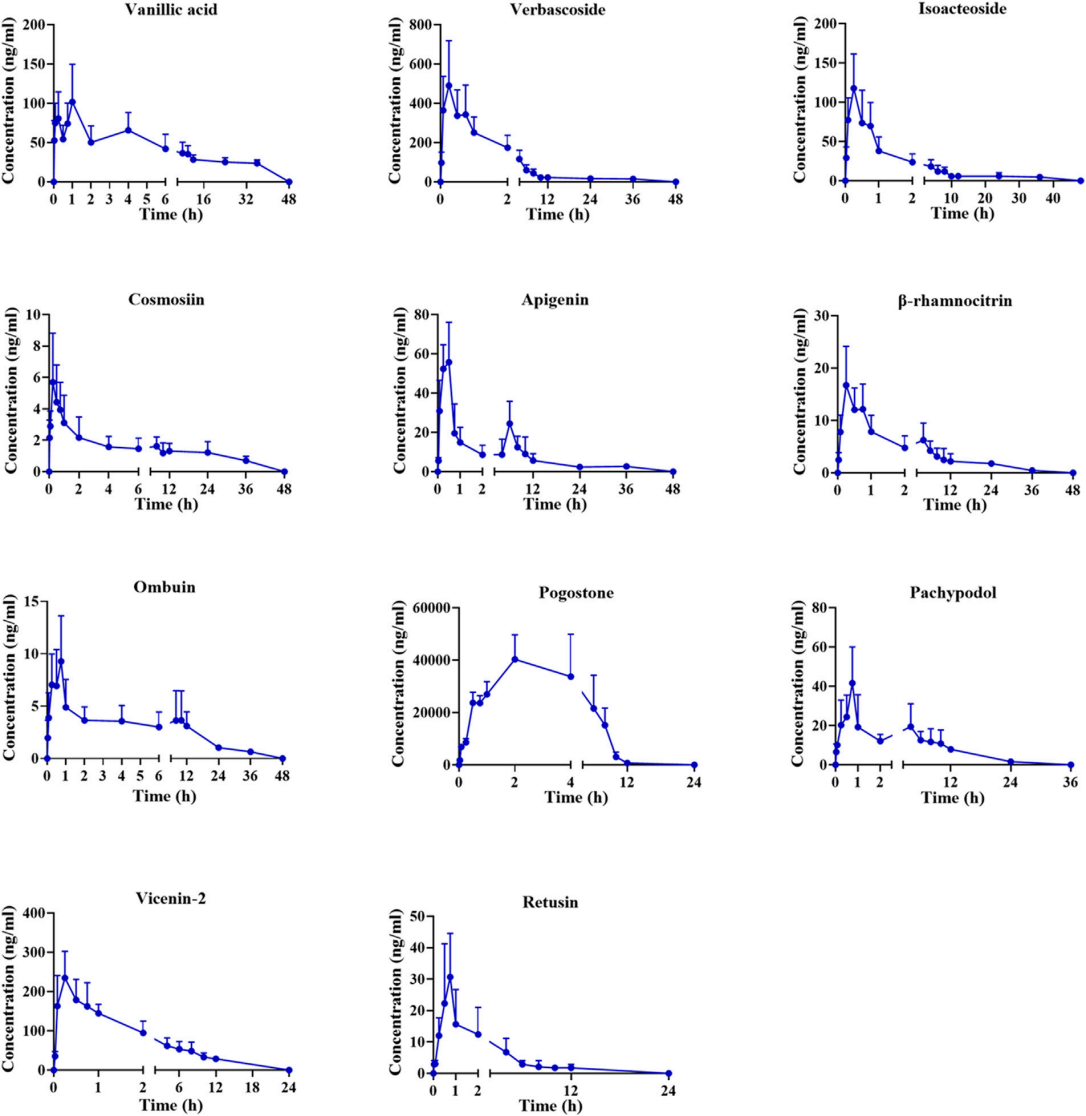
Mean plasma concentration-time profiles in rats after oral administration of PC extract.

Following oral administration of the PC extract, 15 plasma constituents were analyzed using the validated method. Unfortunately, certain analytes such as vitexin, hyperoside, acacetin, and diosmetin-7-O-β-D-glucopyranoside were only detected in the initial plasma samples, leading to difficulties in obtaining a complete PK profile. As a result, these analytes were not included in our analysis. The mean plasma concentration-time curves for the remaining 11 analytes are displayed in Figure 2, with corresponding PK parameters detailed in Table 5.

**TABLE 5:** Pharmacokinetic parameters of 11 components in rats (*n* = 6).

Compound	T_max_	C_max_	T_1/2z_	AUC _(0-tn)_	AUC _(0-∞)_
	(h)	(μg/L)	(h)	(μg/L*h)	(μg/L*h)
Vanillic acid	1.4 ± 1.4	115.8 ± 36.8	2.5 ± 0.1	1,314.1 ± 172.4	1,314.1 ± 172.4
Verbascoside	0.4 ± 0.3	553.9 ± 159.4	2.6 ± 0.21	1702.2 ± 394.5	1702.2 ± 394.5
Isoacteoside	0.5 ± 0.3	109.3 ± 41.5	2.9 ± 0.3	383.8 ± 93.8	383.8 ± 93.8
Cosmosiin	0.5 ± 0.3	6.2 ± 2.5	3.7 ± 0.6	50.1 ± 10.2	50.1 ± 10.2
Apigenin	0.3 ± 0.2	64.2 ± 15.8	2.7 ± 0.6	254.7 ± 65.5	254.7 ± 65.5
β-rhamnocitrin	0.5 ± 0.3	17.4 ± 4.5	4.8 ± 2.6	111.9 ± 37.7	112.7 ± 38.4
Ombuin	0.6 ± 0.2	11.2 ± 1.5	3.8 ± 1.8	95.1 ± 28.1	95.4 ± 28.4
Pogostone	1.9 ± 1.3	37204.0 ± 10,901.0	0.8 ± 0.5	258294.0 ± 93,104.7	258320.9 ± 93120.6
Pachypodol	0.8 ± 0.2	47.8 ± 15.9	2.0 ± 0.1	243.4 ± 63.5	243.4 ± 63.5
Vicenin-2	0.3 ± 0.1	249.4 ± 50.8	1.2 ± 0.6	968.0 ± 131.9	968.8 ± 132.9
Retusin	0.9 ± 0.6	33.0 ± 14.6	1.5 ± 0.2	92.2 ± 33.0	92.2 ± 33.0

The concentration maximum (T_max_) of vanillic acid, verbascoside, isoacteoside, cosmosiin, apigenin, β-rhamnocitrin, ombuin, pogostone, pachypodol, vicenin-2, and retusin were 1.4, 0.4, 0.5, 0.5, 0.3, 0.5, 0.6, 1.9, 0.8, 0.3 and 0.9 h, respectively. The T_max_ of all components was within 2 h, indicating that the absorption of these compounds happened quickly. Pogostone was rapidly absorbed in rats after oral administration, potentially attributed to its low polarity profile and small molecule size (Liu et al., 2012; Chen et al., 2013). Verbascoside and isoacteoside could be rapidly absorbed *in vivo*, which might be related to their relatively large polarity, and previous research has shown comparable PK characteristics. (Zheng et al., 2015). The plasma half-life (T_1/2_) of pogostone was 0.8 ± 0.5 h, which was relatively short compared to other compounds. It is speculated that this might be due to its distribution and elimination rapidly in rats (Zheng et al., 2015). The area-under-the-curve (AUC) for pogostone significantly exceeded that of the other constituents, suggesting that pogostone exhibited a higher plasma exposure level, correlating with its abundant PC content. Additionally, the concentration maximum (C_max_) of pogostone was 37204.0 ± 10,901.0 ng/mL, coupled with its substantial exposure *in vivo*, suggesting that pogostone may be the mian active component in the PC extract (Li et al., 2021). Furthermore, the maximum concentration (Cmax) of pogostone was measured at 37204.0 ± 10,901.0 ng/mL.

The apigenin, *β*-rhamnocitrin, and pachypodol have a double-peak phenomenon in Figure 2, which is likely due to enterohepatic circulation (Wang et al., 2014). Additionally, the absorption of drugs in the gastrointestinal tract is a complex process that is influenced by many physical, chemical, and physiological factors. There are multiple absorption sites in different parts of the gastrointestinal tract, but due to the different permeability of the inner membrane of the cavity to drugs at different sites, the absorption rates and times differ following oral administration. Consequently, absorbed drugs overlap in the blood, creating a bimodal phenomenon (Zhou, 2003).

PK is an indispensable strategy for understanding the behavior *in vivo* after drug administration, which is of great significance in elucidating the mechanism of action, reducing toxic and side effects, optimizing the drug administration program, and guiding the clinical application of drugs (Jin et al., 2019; Liu et al., 2021). Currently, the PK studies of PC mainly focus on a few components, such as pogostone and verbascoside (Li et al., 2012; Chen et al., 2013; Huo et al., 2016). However, research on these individual components cannot reflect the actual PK characteristics of PC after administration. Therefore, in this study, the simultaneous determination of multiple components in rat plasma and PK study by UPLC-MS/MS, which is crucial in elucidating the pharmacological substance basis, mechanism of action, and optimal dosing regimen of PC, and may provide a reference for the clinical application of PC.

Notably, most of the current PK studies are in normal animals, and there are fewer in model animals. Numerous studies have shown that disease states may cause significant alterations in PK parameters (Chen et al., 2022; Liu et al., 2022; Xiong et al., 2023). Since the drugs are mainly used in the pathological state of the body, it is more meaningful to study the PK of TCM in pathological states (Luo et al., 2014). Moreover, PK and pharmacodynamics (PD) are two important interrelated and inseparable aspects in the field of pharmacological research of TCM. PK/PD modeling is extensively utilized in both preclinical and clinical drug research, which aids in gaining a comprehensive and precise understanding of drug effectiveness over time and plasma concentration. Additionally, it provides a valuable reference for optimizing clinical dosage, improving efficacy, and minimizing adverse effects. Therefore, it is vital to explore the ideas and methods of PK-PD modeling of TCM to elucidate the nature and laws (Zhang Z. et al., 2016; Zhu et al., 2023). Only normal rats were investigated in this study, while the PK of PC in model animals was lacking. In the future, The PK research of PC should focus on studies in disease states and develop PK-PD models to elucidate the pharmacodynamic basis and mechanism of action of PC.

## 4 Conclusion

In this study, a UPLC-MS/MS method was developed to simultaneously measure 15 components in rat plasma following the oral administration of PC extract. The method has the benefits of uncomplicated sample preparation and the simultaneous analysis of multiple components within a brief timeframe. Furthermore, the method is specific, stable, and reliable. Moreover, PK results are crucial in elucidating the pharmacodynamic material basis, mechanism of action, and optimal dosing regimen of PC, and may provide guidance for the future advancement and clinical application of PC.

## Data Availability

The original contributions presented in the study are included in the article/Supplementary Material, further inquiries can be directed to the corresponding author.

## Abbreviations

PC, *P. cablin*; TCM, traditional Chinese medicine; IS, internal standard; ME, methanol; ACN, acetonitrile; FA, formic acid; UPLC-MS/MS, ultra-high performance liquid chromatography/tandem mass spectrometry; MRM, multiple reaction monitoring; RE, relative error; RSD, relative standard deviation; LLOQ, lower limits of quantitation; QC, quality control; T_max_, concentration maximum; T_1/2_, plasma half-life; C_max_, concentration maximum; AUC, area-under-the-curve.
