# Supplementary material for: Simultaneous determination of multiple components in rat plasma by UPLC-MS/MS for pharmacokinetic studies after oral administration of Pogostemon cablin extract
Source: Front Pharmacol. 2024 May 22;15:1293464. doi: 10.3389/fphar.2024.1293464 (PMC11150675; doi:10.3389/fphar.2024.1293464)
Supplement: Supplementary file 1 [file Table1.DOCX]

Supplementary Material

**Supplementary Table S1.** Mass spectrometry parameters of 15 components and IS in PC extract.

| Compound | Ion mode | Precursor Ion (*m/z*) | Product Ion (*m/z*) | Fragmentor (V) | Collision Energy (V) |
| --- | --- | --- | --- | --- | --- |
| Vanillic acid | Negative | 167.0 | 152.0 | 98 | 12 |
| Vitexin | Negative | 431.1 | 311.0 | 171 | 20 |
| Verbascoside | Negative | 623.2 | 161.0 | 89 | 36 |
| Isoacteoside | Negative | 623.2 | 160.9 | 184 | 40 |
| Hyperoside | Negative | 463.1 | 300.0 | 146 | 28 |
| Cosmosiin | Negative | 431.1 | 268.0 | 209 | 36 |
| Apigenin | Negative | 269.0 | 117.0 | 146 | 44 |
| β-rhamnocitrin | Negative | 315.1 | 165.0 | 146 | 20 |
| Acacetin | Negative | 283.1 | 268.0 | 136 | 24 |
| Ombuin | Negative | 329.1 | 314.0 | 123 | 16 |
| Pogostone | Negative | 223.1 | 139.0 | 224 | 16 |
| Pachypodol | Positive | 345.1 | 330.1 | 115 | 25 |
| Vicenin-2 | Negative | 593.2 | 353.0 | 227 | 40 |
| Retusin | Positive | 359.1 | 344.1 | 141 | 24 |
| Diosmetin-7-O-β-D-glucopyranoside | Positive | 463.1 | 301.0 | 113 | 20 |
| Icariin (IS) | Positive | 677.3 | 531.2 | 136 | 12 |

**Supplementary Table S2.** Results of content determination in PC extract (n = 3).

| Compound | Content (μg/g) |
| --- | --- |
| Vanillic acid | 159.6 ± 6.0 |
| Vitexin | 42.2 ± 1.0 |
| Verbascoside | 13322.5 ± 71.5 |
| Isoacteoside | 2411.1 ± 73.9 |
| Hyperoside | 33.6 ± 0.8 |
| Cosmosiin | 141.3 ± 2.4 |
| Apigenin | 165.3 ± 2.3 |
| β-rhamnocitrin | 1021.5 ± 12.7 |
| Acacetin | 22.5 ± 0.3 |
| Ombuin | 75.2 ± 1.3 |
| Pogostone | 31080.7 ± 623.6 |
| Pachypodol | 867.7 ± 52.3 |
| Vicenin-2 | 2150.1 ± 22.0 |
| Retusin | 596.5 ± 56.2 |
| Diosmetin-7-O-β-D-glucopyranoside | 4.9 ± 0.1 |
